# Novel use of arterial spin labelling in the imaging of peripheral vascular malformations

**DOI:** 10.1259/bjrcr.20200021

**Published:** 2020-03-24

**Authors:** Sanjeev Ramachandran, Jonathan Delf, Jocelyn Brookes, William Adair, Harjeet Rayt, Matthew Bown, Neghal Kandiyil

**Affiliations:** 1 University Hospitals of Leicester NHS Trust, Infirmary Square, Leicester LE1 5WW, United Kingdom; 2 University College London Hospitals NHS Foundation Trust, 235 Euston Road, London NW1 2BU, United Kingdom

## Abstract

We present a novel use of arterial spin labelling (ASL), a MRI perfusion technique, to assess a high-flow, peripheral vascular malformation (PVM), specifically a large arteriovenous malformation in the left forearm of a 20-year-old female. While there has been experience with ASL in the assessment of intracranial vascular malformations, there has been no known use of ASL in the evaluation of PVMs. We also discuss the potential benefits and limitations of ASL in the imaging of PVMs. The promising results from this case warrant further research on ASL in the investigation of PVMs.

## Introduction

Peripheral vascular malformations (PVMs) represent non-lethal failure of early embryonic vascular differentiation, presenting as a broad spectrum of dysmorphic vascular lesions. The classification system of the International Society for the Study of Vascular Anomalies distinguishes vascular tumours from vascular malformations and further divides these by flow dynamics into high- or low-flow lesions.^1^ Cellular phenotypes of PVMs can be lymphatic, venous, capillary, arterial, arteriovenous or mixed. PVMs produce a spectrum of clinical presentations, ranging from incidental findings to life- or limb-threatening complications.^2^ PVMs may undergo rapid growth as a result of infection and hormonal changes (such as pregnancy) with little understood regarding the underlying physiological and haemodynamic mechanisms.^3^

Although clinical assessment remains an important aspect in diagnosing PVMs, imaging has an increasingly central role in the work-up of such lesions. Arterial spin labelling (ASL) is an MR perfusion technique where water in arterial blood is used as an endogenous freely diffusible tracer. To our knowledge, there has been no published literature on the use of ASL in the evaluation of PVMs. We therefore present a novel use of ASL in the imaging of a PVM.

## Case

**Figure 1.**
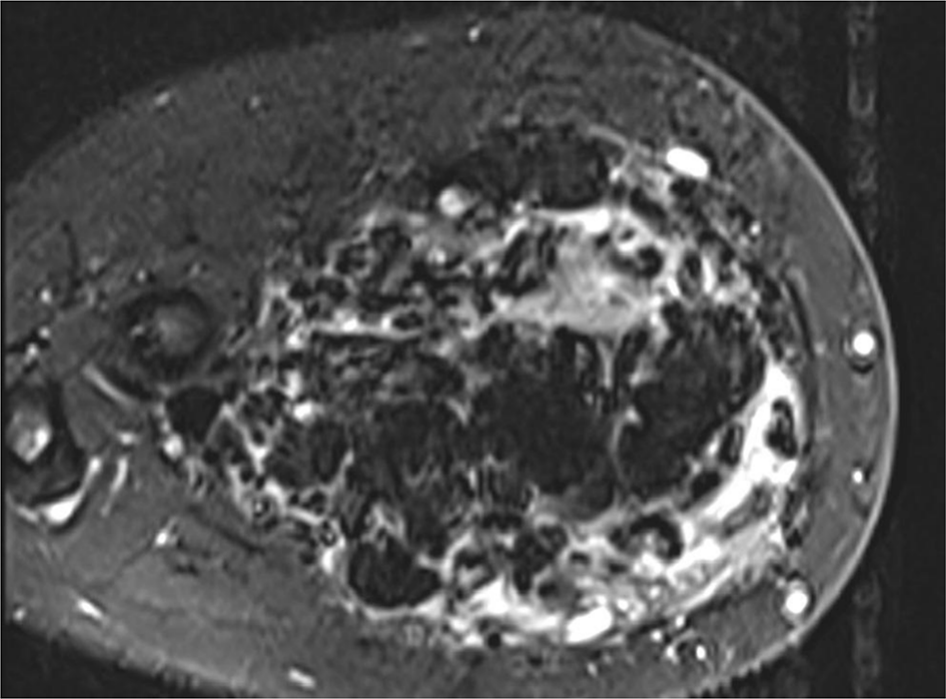
Axial STIR demonstrating a large AVM measuring 8 × 6 × 20 cm in the flexor compartment of the left forearm, extending from proximal to the elbow joint to the distal forearm. This was surrounded by a large volume of connective tissue. No intraosseous involvement is demonstrated. AVM, arteriovenous malformation; STIR, short-tau inversion recovery.

**Figure 2.**
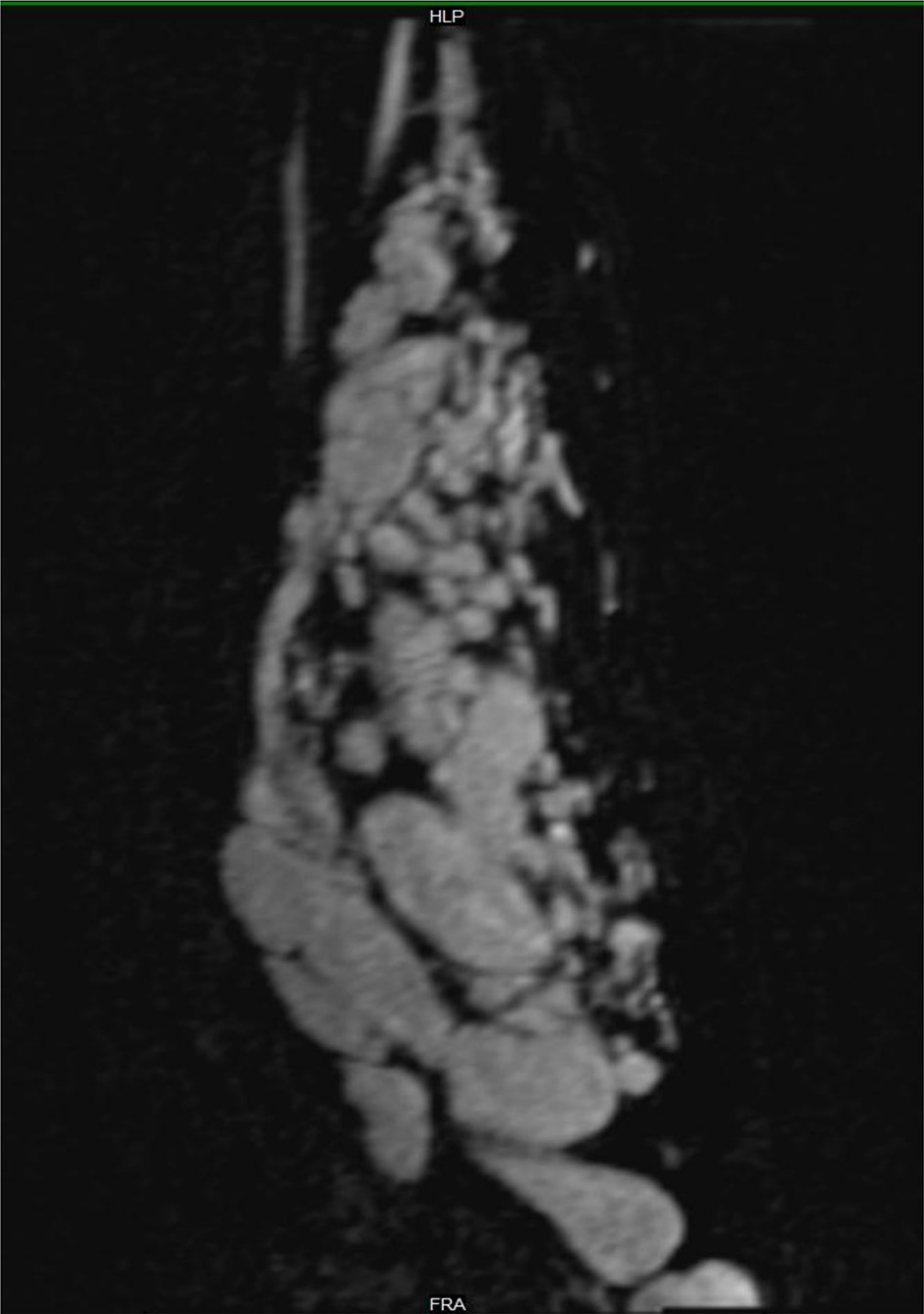
Coronal 3D time-resolved gadolinium enhanced MR angiography (TWIST) demonstrating the presence of an AVM with avid contrast enhancement. The brachial artery is dilated and there are large venous lakes and dilated draining veins are noted in the arm. 3D, three-dimensional; AVM, arteriovenous malformation.

**Figure 3.**
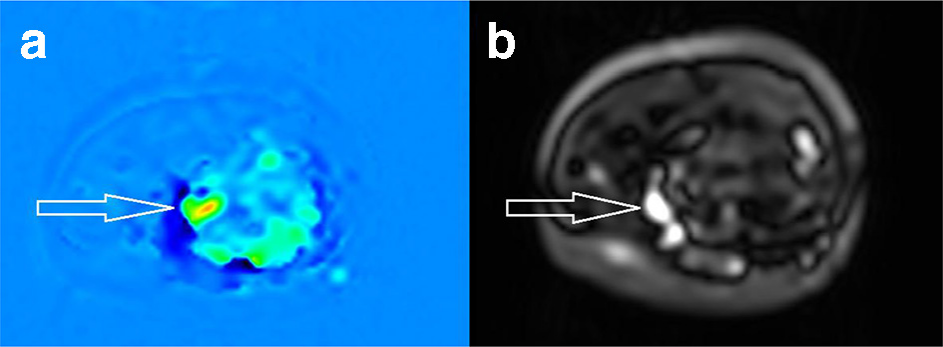
Axial ASL images. (a) RBG image demonstrates perfusion characteristics of labelled inflowing arterial blood, with higher flow indicated by red followed by yellow and green, and no flow indicated by blue. (b) Black/white image presents perfusion with signal intensity proportional to blood flow. Together, these confirm the presence of a high-flow lesion with a focus of maximal flow suggestive of the nidus (white arrows). ASL, arterial spin labelling.

**Figure 4.**
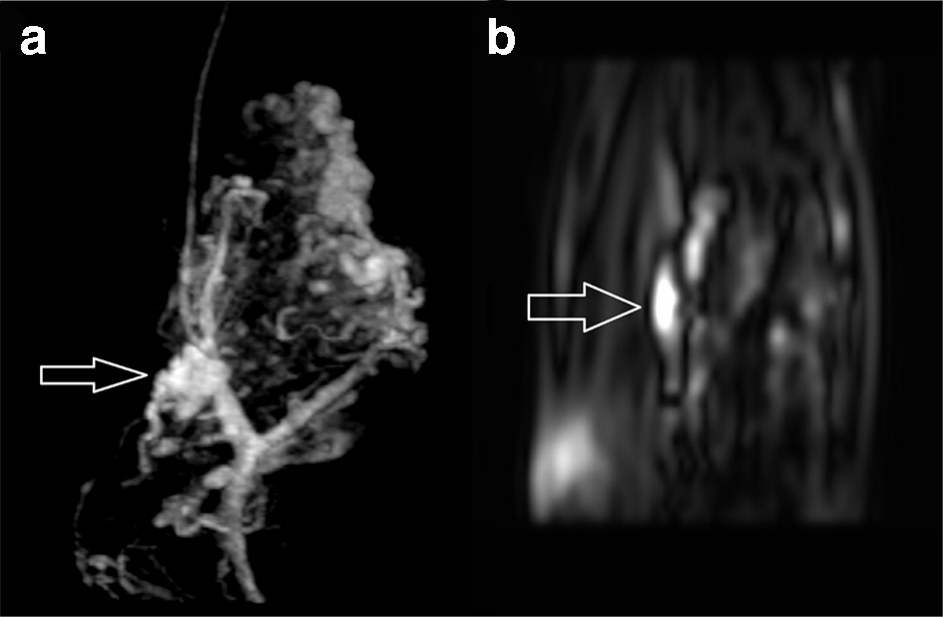
Comparison between coronal TWIST MR angiography (a) and coronal ASL (b) images in approximately the same anatomical plane. A focus of maximal flow identified on ASL correlated with a cluster of vessels demonstrating maximal signal intensity on MR angiography (white arrows), indicative of the nidus. ASL, arterial spin labelling.

A 20-year-old female presents with a swelling in her left forearm. This had been present since the age of 14 years but had significantly grown in size and become increasingly painful during pregnancy. On examination, there was a large, diffuse lesion affecting the majority of the mid and proximal flexor muscle compartment of the left forearm. There was a palpable thrill and tenderness to light touch. There was no evidence of ulceration or digital ischaemia. Doppler ultrasound examination revealed a large cluster of vessels without a well-defined mass. There were both arterial and venous signals present with widespread muscular infiltration. The patient subsequently underwent MRI to further characterise the lesion. The protocols included: T1, T2, short-tau inversion recovery (STIR), three-dimensional time-resolved MR angiography with an intravenous gadolinium bolus (TWIST) and ASL. The conventional MR techniques had demonstrated appearances consistent with 8 × 6×20 cm arteriovenous malformation (AVM) occupying most of the flexor compartment of the forearm with no intraosseus involvement (Figures 1 and 2). ASL had confirmed a high-flow lesion with a focus of maximal blood flow which was suggestive of the nidus (Figure 3). This focus correlated with a region of maximal signal intensity on MR angiography (Figure 4).

ASL imaging was performed prior to gadolinium-enhanced MRA. The labelling slice was applied just above to the elbow joint, where the most proximal extent of the lesion was assessed to be. Pulsed ASL was used with the following parameters: magnetic field strength 1.5 T (MAGNETOM Aera, Siemens Healthcare, Leicester, UK), labelling (bolus) duration 700 ms, post-labelling delay time 1990 ms, flip angle 180^o^, repetition time 4600 ms, echo time 20.44 ms, field of view 240 mm, slice thickness 4 mm, 36 slices, voxel size 1.9 × 1.9×4 mm, relative signal to noise ratio 1.00, base resolution 64 and phase resolution 97%. Image acquisition time was 5 min 36 s. Scrollable greyscale maps of baseline magnetisation were generated using inbuilt software from Siemens, which was used for assessment of possible artefacts and technical error. Mean colour perfusion images were also automatically produced. These images were transferred to the PACS. The maps had full cross-referencing capabilities with three-dimensional reformation to the anatomic MR images.

## Discussion

ASL involves labelling inflowing arterial blood by applying an inversion radiofrequency pulse proximal to the imaging plane, the nature of which varies according to the specific method employed. Labelling methods can be classified as continuous, pseudo continuous, pulsed or velocity-selective.^4^ Subsequently, signal from the arterial blood flow in the imaging plane is subtracted from unlabelled control images to provide a subtracted image. As a result, the signal intensity in the resultant image is proportional to blood flow.^5^ ASL has emerged as a useful tool in the assessment of a range of neurological conditions, including intracranial vascular malformations.^6^

ASL offers several benefits. Firstly, ASL allows us to understand the functional flow dynamics of an AVM and is able to provide an objective, quantitative measure of flow. The differentiation between high- and low-flow lesions is essential to guide management. For example, treatment of high-flow lesions (such as arteriovenous malformations or fistulas) would involve complete occlusion of the nidus or fistulous collection through transarterial embolisation. Conversely, percutaneous sclerotherapy would be offered for low-flow lesions. Indeed, ASL has been shown to be useful in discriminating between cervicofacial vascular malformations in paediatric patients by demonstrating differences in intralesional flow.^7^ Furthermore, it is important to identify high-flow lesions due to their potential to cause systemic complications, such as high output cardiac failure secondary to excessive arteriovenous shunting.^8^ Secondly, identification and characterisation of the nidus on ASL can facilitate planning for endovascular procedures. Thirdly, ASL may be useful in the follow-up of high-flow lesions following treatment, which is supported by studies on intracranial AVMs.^9,10^ Finally, the use of arterial water as an endogenous tracer circumvents the need for intravenous gadolinium (required in most MR angiographic techniques) and its associated risks. As such, ASL can particularly play a role during pregnancy, paediatric patients in the first year of life and in patients with renal impairment.

Conversely, there are technical limitations to ASL in evaluating PVMs, much of which is drawn from previous experience in the imaging of intracranial vascular malformations. To begin, there is recovery of spin inversion in arterial water during its transit, such that the signal reduces by the time it enters venous drainage.^11^ This may impede the assessment of venous drainage beyond the nidus in high-flow lesions. Moreover, the sensitivity of ASL in evaluating lower flow lesions and small calibre arterial feeders and draining veins has not yet been established. Additionally, signal-to-noise ratio, spatial resolution, magnetisation transfer effects and insensitivity to transit time in particular may hinder clinical uptake.^12^ Furthermore, the impact of varying labelling and MR parameters on perfusion measurements and image quality has not yet been fully established. For example, it has been shown that altering parameters such as post-labelling delay time can change nidal, venous and grey matter perfusion in intracranial AVMs.^13^ Total blood flow in high-flow lesions may also be underestimated on ASL, as regions of interests may include not only the high-flow nidus but also the arterial feeders and draining veins, resulting in an average value for flow.^10^ Finally, there is a theoretical risk of signal inhomogeneities in certain vascular territories, depending on the location of the PVM and its arterial supply with regards to the labelling plane. This is a phenomenon that has been demonstrated in intracranial ASL imaging.^14^

To conclude, we present a novel use of a MR perfusion imaging technique to assess PVMs that has provided clinically important information. The promising results from this case warrant further research on ASL in the investigation of PVMs.

## Learning points

- Differentiation between high- and low-flow PVMs is essential in deciding the management, with current imaging work-up including Doppler ultrasound and MRA.
- ASL is an MR perfusion technique where water in arterial blood is used as an endogenous freely diffusible tracer. It has been shown to be useful in assessing intracranial AVMs.
- ASL appears to be a feasible imaging tool in the evaluation of PVMs, and has its own advantages and disadvantages.
